# Mutations in the bacterial cell division protein FtsZ highlight the role of GTP binding and longitudinal subunit interactions in assembly and function

**DOI:** 10.1186/s12866-015-0544-z

**Published:** 2015-10-13

**Authors:** Heidi A. Arjes, Bradley Lai, Ezinwanne Emelue, Adriana Steinbach, Petra Anne Levin

**Affiliations:** Department of Biology, Washington University in St. Louis, St. Louis, MO 63130 USA; Present address: Department of Bioengineering, Stanford University, Stanford, CA 94305 USA

**Keywords:** FtsZ, Cell division, Cytoskeleton, Protein assembly, GTPase activity, FtsZ assembly

## Abstract

**Background:**

Assembly of the tubulin-like GTPase, FtsZ, at the future division site initiates the process of bacterial cytokinesis. The FtsZ ring serves as a platform for assembly of the division machinery and constricts at the leading edge of the invaginating septum during cytokinesis. *In vitro*, FtsZ assembles in a GTP-dependent manner, forming straight filaments that curve upon GTP hydrolysis. FtsZ binds but cannot hydrolyze GTP as a monomer. Instead, the active site for GTP hydrolysis is formed at the monomer-monomer interface upon dimerization. While the dynamics of GTP hydrolysis and assembly have been extensively studied *in vitro*, significantly less is known about the role of GTP binding and hydrolysis *in vivo. ftsZ84*, a GTPase defective allele of *Escherichia coli ftsZ,* provides a striking example of the disconnect between *in vivo* and *in vitro* FtsZ assembly.

**Results:**

Although *ftsZ84* mutants are defective for FtsZ ring formation and division under nonpermissive conditions, they are near wild type for ring formation and division under permissive conditions. *In vitro,* however, purified FtsZ84 is defective in GTP binding, hydrolysis and assembly under standard reaction conditions. To clarify the nature of the FtsZ84 assembly defect, we isolated and characterized three intragenic suppressors of *ftsZ84*. All three suppressor mutations increased the apparent affinity of FtsZ84 for GTP, consistent with improved subunit-subunit interactions along the longitudinal interface. Although kinetic analysis indicates that the suppressor mutations increase the affinity of FtsZ84 for GTP, all three exhibit reduced rates of GTP hydrolysis and fail to support assembly *in vitro*.

**Conclusion:**

Together, our data suggest that FtsZ, and potentially other enzymes whose assembly is similarly regulated, can compensate for defects in catalysis through increases in substrate binding and subunit-subunit interactions. In addition, these results highlight the dichotomy between commonly used *in vitro* assembly conditions and FtsZ ring formation in the complex intracellular milieu.

**Electronic supplementary material:**

The online version of this article (doi:10.1186/s12866-015-0544-z) contains supplementary material, which is available to authorized users.

## Background

Assembly of the tubulin-like GTPase FtsZ at the future division site is a fundamental step in bacterial cytokinesis [1, 2]. In response to an unidentified cell cycle signal, FtsZ transitions from primarily cytoplasmic monomers and/or short polymers into the longer polymers that constitute the FtsZ ring and serve as the foundation for assembly of the division machinery [2]. The FtsZ ring is highly dynamic, with subunit turnover rates on the order of seconds [2]. Although subunit turnover is high, the ring itself is present for ~80 % of the division cycle under nutrient rich conditions [3, 4]. Why the ring remains in place so long is unclear, although studies of cells defective in assembly of downstream components of the division machinery suggest a maturation process may be at work [5, 6]. At the end of the division cycle, in response to another unidentified signal, the FtsZ ring constricts at the leading edge of the invaginating septum. Work from our laboratory and others indicates that FtsZ concentration is constant throughout the cell cycle and that the precise spatial and temporal regulation of bacterial cell division is governed by tightly orchestrated changes in FtsZ assembly dynamics [3, 4, 7].

**Fig. 1:**
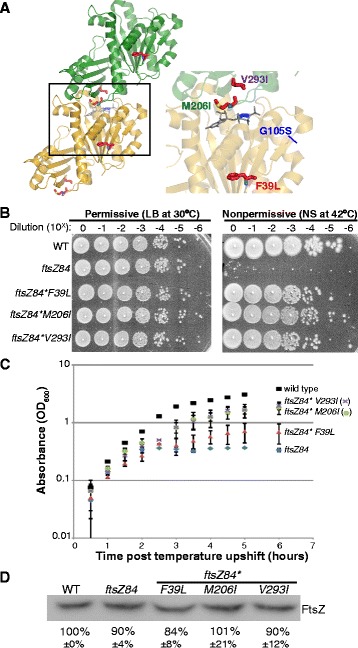
Secondary mutations restore temperature resistance to *ftsZ84* cells *without* increasing FtsZ concentration. **a** Location of the FtsZ84 (G105S) point mutation and the three intragenic suppressors of that mutation mapped onto an FtsZ dimer from *Staphylococcus aureus*. G10S5S (blue) is the original *ftsZ84* point mutation. GDP is shown in gray. The three intragenic suppressors F39L, M206I and V293I are highlighted in the inset. The crystal structure is modified from the *S. aureus* FtsZ dimer structure (PDB ID: 3WGN) [52]. **b** Plating efficiency assays of wild-type, *ftsZ84*, and the three intragenic *ftsZ84* suppressor strains. Tenfold dilutions of cells cultured in permissive conditions were plated under permissive and nonpermissive conditions. This experiment was repeated 3 times with identical results. One representative experiment is shown. **c** Growth as measured by absorbance (OD_600_) of wild-type, *ftsZ84*, and the three intragenic *ftsZ84* suppressor strains under nonpermissive conditions. Error bars represent standard deviation of 3 independent experiments. **d** Quantitative immunoblot indicates that intracellular FtsZ concentrations are wild type in *ftsZ84* and ftsZ84* mutants under nonpermissive conditions. After ~3 mass doubling periods in LB no salt, cells were sampled at equivalent OD’s to ensure the same amount of protein was loaded per lane and protein bands were normalized to total protein (Ponceau staining) as a loading and transfer control. ImageJ software was used to quantify band intensity. The average and standard deviation of 3 independent experiments are shown below the blot

Like tubulin, multimerization is a prerequisite for FtsZ-mediated GTP hydrolysis. FtsZ binds to GTP as a monomer but cannot hydrolyze it as such. The active site for GTP hydrolysis is formed at the longitudinal interface between two FtsZ subunits where the GTP-binding pocket of one monomer contacts the T7 synergy loop of the adjacent monomer (Fig. 1a) [2, 8]. *In vitro*, GTP binding stimulates FtsZ assembly into single stranded polymers (also known as protofilaments). Higher order assembly of FtsZ protofilaments into polymer bundles is also possible *in vitro*, depending on buffer conditions, and, in some cases, the bacterial species from whence the FtsZ was derived [9, 10]. GTP-bound FtsZ protofilaments are initially straight, curving only upon nucleotide hydrolysis [2]. Once GTP has been hydrolyzed to GDP, polymers can disassemble and exchange GDP for GTP and assemble once again [2].

Although GTP binding and hydrolysis have a strong impact on FtsZ assembly *in vitro,* their role *in vivo* is less clear. The disconnect between *in vitro* and *in vivo* data is exemplified by *ftsZ84*, a heat-sensitive allele of *ftsZ* encoding a point mutation in the GTP binding pocket (G105S) [11]. While *ftsZ84* cells exhibit predominantly normal FtsZ ring formation and division under permissive conditions (30 °C in LB with 1 % NaCl), under nonpermissive conditions (42 °C in LB with no salt), *ftsZ84* mutants are defective for FtsZ ring formation and division [12, 13]. Surprisingly, although functional *in vivo* under permissive conditions, FtsZ84 protein is unable to assemble into protofilaments *in vitro*, regardless of temperature, and is severely defective in GTP binding and hydrolysis [14–16]. The inability of FtsZ84 to assemble *in vitro* when it can support division *in vivo* under permissive conditions raises questions about the relationship between *in vitro* FtsZ assembly and *in vivo* function. Differences between *in vitro* and *in vivo* assembly are likely due in part to limitations in our ability replicate the *in vivo* environment *in vitro;* not only in regard to buffer conditions, but also in regard to the host of modulatory proteins that are normally found in the cellular milieu. With regard to the latter, ZapA in particular has been found to promote lateral interactions and assembly of FtsZ *in vitro* [17].

To clarify the nature of the *ftsZ84* defect at the functional level*,* we identified three intragenic suppressors that restored FtsZ ring formation and division to *ftsZ84* mutants under nonpermissive conditions and GTP binding, but not polymerization, *in vitro*. Together our findings highlight a fundamental role for robust longitudinal subunit interactions in assembly of FtsZ protofilaments and suggest these interactions are reinforced *in vivo* through the actions of multiple FtsZ modulatory proteins.

## Results

### Identification of intragenic suppressors of *ftsZ84*

To identify spontaneous suppressor mutations that restore the ability of *ftsZ84* mutants to grow under nonpermissive conditions, we cultured *ftsZ84* cells to mid-exponential phase (OD_600_ = 0.2-0.5) under permissive conditions (30 °C, LB 1 % NaCl), then washed and plated cells on no salt medium at 42 °C overnight. Under these restrictive conditions, *ftsZ84* mutants were reduced more than 10^5^-fold for colony formation (Fig. 1b). Any colonies that arose after overnight incubation were the result of spontaneous suppressor mutations. Suppressors were isolated at a frequency of 1.5*10^-5^_,_ consistent with the majority representing loss-of-function mutations in average size (~1 kb) genes. We also employed ethyl methanesulfonate (EMS) mutagenesis in a second screen in an attempt to identify additional intragenic suppressor mutations. EMS alkylates guanine and commonly causes thymine to pair with the alkylated guanine residues causing G:C to A:T substitutions.

To specifically identify those suppressor mutations that were intragenic, we employed P1 phage transduction to determine the linkage between individual mutations and the *ftsZ84* allele [8, 9, 15, 18]. Briefly, we transduced genomic DNA from the suppressors into wild-type *E. coli* and selected for tetracycline resistance (the *ftsZ84* mutation is 55 % linked to a tetracycline resistant (TetR) Tn10 insertion in the *leuD* gene) [18]. The resulting colonies were analyzed for temperature sensitivity (denoting the suppressor mutation *was not* linked to *ftsZ84*) or temperature resistance (denoting the suppressor mutation *was* linked to *ftsZ84*).

7 of 96 spontaneous suppressor mutations and 10 of 14 EMS generated suppressor mutations were linked to *ftsZ* by P1 transduction. Sequencing revealed that all linked mutations were secondary mutations within the *ftsZ84* coding sequence. The remaining 93 suppressor mutations did not co-transduce with *ftsZ84* suggesting they are located in other chromosomal regions distal to *ftsZ* and the Tn*10*::*tetR* marker.

**Fig. 2:**
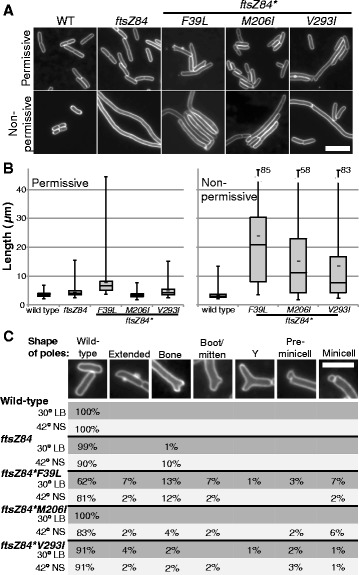
ftsZ84* suppressor mutants exhibit variable cell lengths and polar shapes under permissive and nonpermissive conditions. **a** Representative images of cell membrane staining of live wild-type cells, *ftsZ84* and ftsZ84* strains under permissive and nonpermissive conditions. Bar = 5 μm. **b** Box and whisker plots of cell length distributions in permissive or nonpermissive conditions. Cells were grown in permissive conditions to an OD_600_ of 0.2-0.4 and sampled. For the nonpermissive conditions, cells were then backdiluted to an OD_600_ of 0.05 and grown to a sampling OD_600_ of 0.2 to 0.4. The box indicates the middle 50 % of values, the line indicates the median, the short bar indicates the mean, and the whisker bars represent the span of data in the lowest quartile (below the box) and the highest quartile (above the box). Values for ftsZ84*F39L and ftsZ84*V293I are conservative as many filaments extended past the field of view and could not be quantified. Note the upper values for the ftsZ84* mutants have been truncated; the number indicates the highest value measured. For permissive conditions, n = 146 (wild type), 132 (*ftsZ84*), 138 (ftsZ84*F39L), 139 (ftsZ84*M206I) and 110 (ftsZ84*V293I). For nonpermissive conditions, n = 102 (wild type), 42 (ftsZ84*F39L), 83 (ftsZ84*M206I), and 129 (ftsZ84*V293I). **c** ftsZ84* suppressor mutants exhibit branching, abnormal polar morphologies, and minicell formation. The fraction of cells exhibiting each morphology was determined by analyzing ~100-150 cells, the only exceptions being *ftsZ84*, ftsZ84*F39L, and ftsZ84*M206I under nonpermissive conditions where fewer cells were examined, respectively, as only whole cells were scored and it was rare to visualize an entire cell in the frame in these samples. For permissive conditions, n = 146 (wild type), 132 (*ftsZ84*), 138 (ftsZ84*F39L), 139 (ftsZ84*M206I), and 110 (ftsZ84*V293I). For nonpermissive conditions, n = 102 (wild type), 10 (*ftsZ84*), 42 (ftsZ84*F39L), 83 (ftsZ84*M206I) and 129 (ftsZ84*V293I). Bar = 3 μm

We isolated three different intragenic suppressor mutations within the *ftsZ* open reading frame: a T to C transition at base pair 115 that changes a phenylalanine to a leucine (F39L), a G to A transition at base pair 618 that changes a methionine to an isoleucine (M206I) and a G to A transition at base pair 877 that changes a valine to an isoleucine (V293I) (Fig. 1a). For simplicity, all intragenic suppressors are designated ftsZ84* to indicate the presence of the original G105S mutation and a second intragenic suppressor mutation. The F39L mutation is located in the core of FtsZ and presumably impacts the structure of the active site (Fig. 1a). ftsZ84*F39L is phenotypically the weakest *ftsZ84* suppressor with regard to growth rate and cell size under nonpermissive conditions (Fig. 1c, 2)*.* By contrast, M206I and V293I are nearer to wild type with regard to both growth and cell size under the same conditions. M206I is located in the T7-synergy loop, a region that contacts the GTP binding site in adjacent monomers and is essential for GTP hydrolysis (Fig. 1). ftsZ84*M206I cells restore length approximately twice as well as F39L at nonpermissive conditions (Fig. 2). V293I is located on the longitudinal subunit-subunit interface, and like M206I, presumably stabilizes interactions between monomers in FtsZ protofilaments (Fig. 1). Like ftsZ84*M206I mutants, ftsZ84*V293I cells restore cell length under nonpermissive conditions (Fig. 2).

Although we screened ~10^8^ cells in 5 independent screens (4 screens for spontaneous suppressors and 1 EMS screen), we repeatedly isolated the same three intragenic suppressors of *ftsZ84*. In screens for spontaneous suppressor mutations we isolated the F39L mutation twice, the M206I mutation 3 times and the V293I mutation twice. Using EMS, we isolated M206I once and identified 9 independent V293I mutations. (The nature of EMS mutagenesis precluded the identification of F39L, which requires a T to C transition.) Identification of such a limited number of suppressors is consistent with a strong relationship between primary sequence and FtsZ function *in vivo*.

Notably, transduction of the three ftsZ84* alleles – ftsZ84*F39L, ftsZ84*M206I, and ftsZ84*V293I – into a fresh MG1655 wild-type background indicated that all three intragenic suppressor mutations are sufficient to fully restore colony forming ability under nonpermissive conditions. All the intragenic suppressor mutants displayed wild-type colony forming ability and colony size under permissive conditions (Fig. 1b). The colony-forming ability of all three suppressor-bearing strains was more than 10,000-fold higher than the *ftsZ84* parent strain under nonpermissive conditions (Fig. 1b). ftsZ84* suppressor colonies appeared to be slightly smaller than those of the wild-type strain, but were otherwise morphologically normal (Fig. 1b).

### Whole genome sequencing of *ftsZ84* revealed linked mutations in *ftsW* and *murE*

In the course of sequencing suppressor mutations, we identified 2 additional mutations located between *ftsZ84* and the *tetR* marker that differed from the MG1655 parent. These point mutations, in *ftsW* and *murE* respectively*,* were inseparable from the *ftsZ84* mutation by P1 transduction. The tight linkage between all three genes (*ftsZ84, ftsW,* and *murE*) suggests that these mutations are the consequence of the N-nitrosoguanidine mutagenesis used to generate the original *ftsZ84* (PAT84) strain [12, 19].

FtsW is an integral membrane protein that stabilizes the FtsZ ring and recruits FtsI (PBP3) to the division site [2, 20, 21]. The guanine to adenine mutation at residue 44 replaces a glycine with glutamic acid in the first transmembrane domain (G15E). Given its essential nature, we wondered if insertion of the charged glutamic acid residue in the first hydrophobic transmembrane domain of FtsW by virtue of the G15E mutation might reduce its potential to be accurately inserted in the membrane and contribute to the heat sensitivity of *ftsZ84* cells. However, expression of wild-type *ftsW* from a plasmid in the *ftsZ84* background (HA223) did not complement the heat sensitivity of *ftsZ84* cells, suggesting the *ftsW* G15E mutation does not play a role in the conditional *ftsZ84* phenotype (data not shown).

MurE is a cytoplasmic protein that catalyzes the addition of mesodiaminopimelate to peptidoglycan monomers [22]. The guanine to adenine mutation at position 1381 in *murE* changes a valine to an isoleucine in a poorly conserved region 34 residues from the C-terminus (V461I). In addition to the V461I mutation, there is also a synonymous mutation at base pair 1113, G1113A, which is in the wobble position and encodes for the correct amino acid (alanine) at position 371. Due to the conservative nature of the V461I mutation in MurE, we did not determine if exogenous *murE* expression suppressed the *ftsZ84* phenotype.

### Intragenic suppressors grow more slowly than wild-type cells under nonpermissive conditions

Despite being near wild type for colony-formation, all three intragenic suppressor mutants exhibited detectable reductions in growth rate in liquid medium under nonpermissive conditions as compared to wild-type cells (Table 1). Wild-type MG1655 *E. coli* had a mass doubling time of 26.2 minutes in no salt media at 42 °C and *ftsZ84* cells grew at approximately the same rate, increasing exponentially under nonpermissive conditions for ~4 doubling periods before growth arrested. This increase in mass for several generations prior to a rapid growth arrest parallels what we have observed in *B. subtilis* cells subjected to a prolonged block in division (Fig. 1c, Table 1) [23]. Consistent with their reduced colony size, all three intragenic suppressor strains exhibited reduced rates of mass increase under nonpermissive conditions. Of the three intragenic suppressors, ftsZ84*F39L exhibited the largest growth defect, taking more than twice the time of wild-type MG1655 cells to double in mass (~57 minutes) (Fig. 1c, Table 1). ftsZ84*M206I and ftsZ84*V293I cells grew more robustly, albeit slower than their wild - type counterparts (mass doubling times ~34 and 38 minutes, respectively) (Fig. 1c, Table 1). We observed no detectable difference in mass doubling time between strains under permissive conditions (Table 1).

**Table 1:** Doubling times of ftsZ84* strains. Strains were grown in permissive conditions (LB at 30 °C) or nonpermissive conditions (LB-no salt at 42 °C) and doubling times during exponential phase were calculated

	Permissive Doubling Time (min)	Nonpermissive Doubling Time (min)
WT	49.4 (±5.2)	26.2 (±2.8)
ftsZ84	47.6 (±7.1)	na
ftsZ84*F39L	51.5 (±3.1)	57.3 (±2.1)
ftsZ84*M206I	51.4 (±2.7)	33.6 (±3.2)
ftsZ84*V293I	49.0 (±4.1)	38.4 (±3.3)

Importantly, all three intragenic suppressors had little impact on the intracellular concentration of FtsZ84 (Fig. 1d). On quantitative immunblots probed with antisera generated against *E. coli* FtsZ (the gift of David Weiss), FtsZ84 levels were ~90 % of wild-type levels and the levels of all mutants were similar to FtsZ84 or wild-type levels (FtsZ84*F39L was 84 % of wild-type, FtsZ84*M206I was 101 % of wild-type levels and FtsZ84*V293I was 90 % of wild-type levels) (Fig. 1d). These data indicate that all three mutations suppress *ftsZ84* associated heat sensitivity by altering FtsZ-ring assembly or maturation dynamics rather than elevating FtsZ levels, which has previously been shown to suppress *ftsZ84* [24, 25].

### Intragenic suppressors partially rescue *ftsZ84* filamentation and FtsZ ring formation under nonpermissive conditions

While the intragenic suppressor mutations restored growth and colony formation to *ftsZ84* mutants under nonpermissive conditions, all three ftsZ84* strains exhibited increases in cell length, consistent with retaining a partial defect in cell division. Under nonpermissive conditions (LB-no salt medium at 42 °C), wild-type MG1655 *E. coli* averaged ~3.4 μm in length (range = 2 to 13 μm). *ftsZ84* cells were extensively filamentous, as previously reported (Fig. 2) [12]. (Precise length could not be determined, as the majority of *ftsZ84* filamentous cells extended beyond the field of view.) Of the ftsZ84* mutants, ftsZ84*F39L mutants exhibited the most filamentous phenotype under nonpermissive conditions with an average length of 24 μm (range = 3.5 to 85 μm) (Fig. 2). (Because very long filaments extended out of the field of view, the calculated average is likely to be a significant underestimate.) Although ftsZ84*M206I and ftsZ84*V293I mutants rescued the cell division phenotype to a greater extent than ftsZ84*F39L, both strains exhibited increases in cell size with average lengths of ~15 μm (range 1.7 to 58 μm) and 13 μm (range 2.2 to 83 μm) respectively (Fig. 2).

**Fig. 3:**
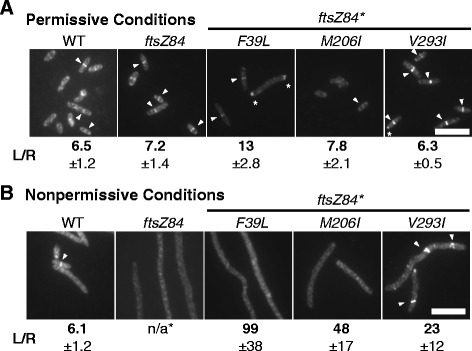
ftsZ84* suppressor mutations restore FtsZ ring formation to varying degrees under nonpermissive conditions. Representative images of cells grown under (**a**) permissive conditions or (**b**) nonpermissive conditions and labeled for cell wall and FtsZ. Arrowheads indicate FtsZ rings. Bar = 5 μm. The length per ring (L/R) ratio was calculated by dividing the total length of all cells by the number of FtsZ rings counted. Average and standard deviation of 3 biological replicates is shown below each image

As expected based on their ability to suppress *ftsZ84* associated lethality, all three intragenic suppressors restored FtsZ ring formation to ftsZ84* cells cultured under nonpermissive conditions. In contrast to *ftsZ84* cells which had no observable rings (length/ring (L/R) ratio >400 μm), ftsZ84*M206I and ftsZ84*V293I exhibited L/R ratios of 48 μm and 23 μm respectively (Fig. 3b). Consistent with its highly filamentous nature ftsZ84*F39L mutants had very few FtsZ rings under nonpermissive conditions (L/R ratio ~100 μm) (Fig. 3b). For reference, wild-type cells exhibited an L/R ratio of 6.1 μm in LB-no salt at 42 °C in (Fig. 3).

### ftsZ84*F39L and ftsZ84*V293I mutants exhibit increases in cell length under permissive conditions

Intriguingly, two of the ftsZ84* intragenic suppressor mutants, ftsZ84*F39L and ftsZ84*V293I, exhibited significant increases in cell length under permissive conditions, suggesting persistent defects in FtsZ assembly. Under permissive conditions, wild-type cells averaged ~3.5 μm in length with a cell length distribution between 2.1 and 6.8 μm, in agreement with previous reports [26] (Fig. 2). Both the *ftsZ84* parent strain and ftsZ84*M206I exhibited near wild-type lengths and were essentially wild type with regard to size under permissive conditions, consistent with near normal rates of division (~4.3 μm, distribution 2.5 to 15 μm and ~3.4 μm, distribution 1.8 to 7.7 μm respectively) (Fig. 2). In contrast to both the *ftsZ84* parent and ftsZ84*M206I, under permissive conditions ftsZ84*F39L and ftsZ84*V293I cells were significantly longer than wild type, suggesting a persistent defect in division. ftsZ84*F39L mutants had a wide distribution of lengths (3.8 to 44 μm) with an average of 7.7 μm (Fig. 2). While somewhat less filamentous, ftsZ84*V293I cells were ~40 % longer than wild-type cells and exhibited a size distribution similar to the *ftsZ84* parent (2.4 to 15 μm) under permissive conditions (Fig. 2).

The L/R ratios of the suppressor strains did not always track with their length distributions under permissive conditions, suggesting that at least a subset of the ftsZ84* mutants are defective in aspects of division subsequent to FtsZ ring formation. Wild-type cells, *ftsZ84* mutants, and ftsZ84*M206I cells exhibited L/R ratios of ~ 6.5 μm, ~7.2 μm, and ~7.8 μm, respectively (Fig. 3a). While ftsZ84*F39L cells exhibited an elevated L/R ratio of 13 μm under permissive conditions, consistent with their filamentous nature, ftsZ84*V293I mutants exhibited a wild-type L/R ratio (~6.3 μm) under permissive conditions despite their large distribution of cell sizes (Fig. 2, 3a). This discrepancy between cell length distribution and L/R ratio suggests that while FtsZ84*V293I is capable of assembly under permissive conditions, FtsZ84*V293I rings are impaired for function.

### ftsZ84* suppressor mutants exhibit abnormal polar morphologies

Work from the Young laboratory indicates that defects in FtsZ ring orientation and/or structure lead to the formation of polar extensions, minicells, and Y-shaped branched cells – the result of aberrant insertion of inert peptidoglycan (normally only inserted at cell poles) [27]. Consistent with defects in FtsZ localization and/or cytokinetic ring integrity, we occasionally observed abnormal polar morphology and cell branching in ftsZ84* mutants. ftsZ84*F39L mutants exhibited the most severe polar phenotypes with ~40 % and ~20 % of cells displaying abnormal polar phenotypes (branching, polar extensions, and minicells) under permissive and nonpermissive conditions respectively (Fig. 2c). The other mutants, ftsZ84*M206I and ftsZ84*V293I exhibited wild-type or near wild-type FtsZ localization under permissive conditions (Fig. 2c). However, in both cases we observed a slight increase in polar extensions and/or minicells under nonpermissive conditions (Fig. 2c).

### Intragenic suppressor mutations increase FtsZ84-mediated GTP hydrolysis at standard GTP concentrations

Based on the observation that all three ftsZ84* alleles supported cytokinetic ring formation, we speculated that the intragenic suppressor mutations increase FtsZ84 subunit-subunit affinity – either directly or by enhancing interaction with GTP – to levels sufficient to permit assembly *in vivo* in the presence of stabilizing modulatory proteins but insufficient to allow assembly *in vitro*. To explore this possibility, we evaluated the ability of the FtsZ84* mutants to hydrolyze GTP *in vitro* under conditions standardly employed to assess FtsZ function (pH 6.5, 1 mM GTP) [8, 10, 28]. Because two FtsZ monomers must interact to form the GTP hydrolysis site, GTPase activity is commonly employed as a proxy for FtsZ dimerization potential [8, 28, 29].

Consistent with increases in dimerization affinity, under standard conditions all three FtsZ84* proteins displayed an approximately 6-fold increase in GTPase activity relative to the FtsZ84 (G105S) single mutant. Under standard reaction conditions (50 mM MES, pH 6.5, 50 mM KCl, 2.5 mM MgCl_2_, 1 mM EGTA, 1 mM GTP, 5 μM FtsZ, 30 °C), wild-type FtsZ GTPase activity was 7 GTP/FtsZ/min and FtsZ84 was 0.47 GTP/FtsZ/min (Table 2). GTP hydrolysis rates for the three intragenic suppressors were ~50 % of wild-type FtsZ at ~3 GTP/FtsZ/minute for FtsZ84*F39L, FtsZ84*M206I, and FtsZ84*V293I, respectively (Table 2). The enhanced GTPase activity exhibited by all three FtsZ84* mutants is consistent with relatively robust dimerization potential.

**Table 2:** FtsZ GTPase turnover rates. GTPase assays were carried out in identical conditions to the standard conditions used for light scattering and EM (50 mM MES, pH 6.5, 50 mM KCl, 2.5 mM MgCh, 1nM EGTA, 1 mM GTP, 30 °C). Rates were calculated as GTP consumed/FtsZ subunit/min

FtsZ species	GTP/FtsZ/min	Std. dev.
FtsZ (wild type)	7	±2.28
FtsZ84 (G105S)	0.47	±0.05
FtsZ84*F39L	3.1	±0.58
FtsZ84*M206I	3	±1.29
FtsZ84*V293I	3	±1.06
FtsZ*F39L	0.9	±0.19
FtsZ*M206I	6	±1.50
FtsZ*V293I	3	±1.16

### FtsZ84* mutants restore GTP binding but not catalytic rate to FtsZ84

The position of the intragenic suppressor mutations outside FtsZ’s GTP binding pocket makes it unlikely that the interaction between FtsZ84* monomers and GTP is directly impacted. At the same time, the substantial increase in GTP hydrolysis exhibited by the FtsZ84* mutants suggests that increased subunit-subunit interaction and/or conformational changes compensate for the GTP binding defect associated with the G105S mutation. To investigate this possibility, we generated Michaelis-Menten curves for FtsZ, FtsZ84, and the three FtsZ84* mutants by varying GTP concentration while maintaining FtsZ concentration at 5 μm under our standard buffer conditions (50 mM MES, pH 6.5, 50 mM KCl, 2.5 mM MgCl_2_, 1nM EGTA, 0.25-4.0 mM GTP, 22 °C). At higher concentrations of GTP (>4 mM for wild-type FtsZ) we observed substrate inhibition and these points were excluded from out data set. The GTPase activity of FtsZ has previously been shown to follow Michaelis-Menten kinetics by two groups using different buffer conditions [30, 31].

**Fig. 4:**
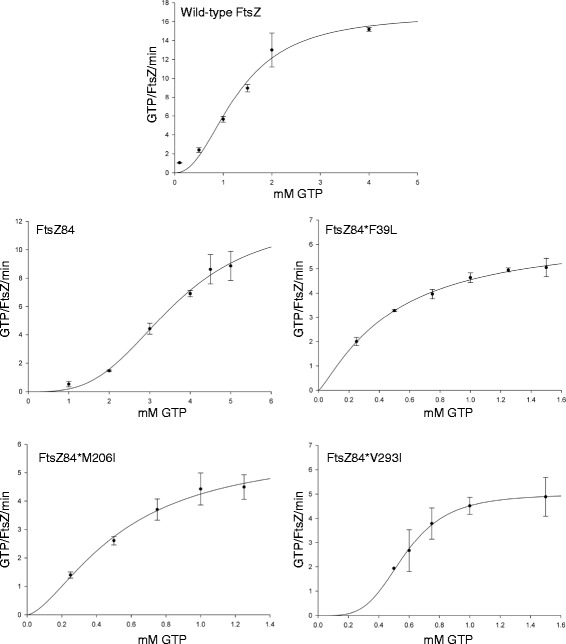
Steady state kinetic analysis of FtsZ, FtsZ84, and FtsZ84* proteins. GTPase assays were carried out as described in materials and methods (50 mM MES, pH 6.5, 50 mM KCl, 2.5 mM MgCl_2_, 1 mM EGTA). FtsZ concentration was kept constant at 5 μM and GTP concentration were varied from 0.1 μM to 5 μM (different ranges were used depending on the FtsZ mutant analyzed). The best fit for our data was obtained using a Hill-modified Michaelis-Menten equation using Sigma Plot software. K_m_ and V_max_ values are listed in Table 3

To determine K_m_ and V_max_ for all five FtsZ variants, we fit the GTPase activity data to a Hill-modified Michaelis-Menten equation, as that provided the best fit for our data (Fig. 4). K_m_ provides an estimate of the disassociation constant (K_d_) which correlates inversely with the affinity of the interaction between FtsZ and GTP. V_max_ is a reflection of the maximal rate of catalysis. Although interpretation is not as straightforward as it might be due to FtsZ’s nature as a polymerizing enzyme, *n* is a measure of cooperative binding (*n* <1 indicates cooperitivity).

K_m_ data suggest that the amino acid substitutions in the FtsZ84* mutants compensate for the impact of the G105S mutation by enhancing interactions between FtsZ and GTP. Wild-type FtsZ had a K_m_ of 1.3 mM, similar to the value obtained by Salvarelli et al. (0.3 mM) but much higher than the Km of 82 μM reported by Sossong *et al.* [30, 31]*.* Differences between our data and that of Sossong *et al.* can potentially be attributed to the different reaction conditions used in their study [30, 31]. In contrast to wild-type FtsZ, FtsZ84 exhibited a K_m_ of 3.6 mM (Table 3). The 3-fold increase between the K_m_ of wild-type FtsZ and the K_m_ of FtsZ84 is consistent with previous work indicating that FtsZ84 is defective in GTP binding [14, 15]. All three FtsZ84* mutants exhibited K_m_ values approximately seven-fold less than FtsZ84, supporting the idea that the secondary mutations enhance interactions between FtsZ84 and GTP (Table 3). FtsZ84*F39L, FtsZ84*M206I and FtsZ84*V293I displayed K_m_ values ranging from 0.5-0.6 mM (Table 3).

**Table 3:** Steady state kinetic analysis of FtsZ, FtsZ84 and FtsZ84*. GTPase assays were carried out in identical conditions as the standard GTPase assay (Table 2) except GTP concentration was varied and the reactions were performed room temperature (50 mM MES, pH 6.5, 50 mM KCl, 2.5 mM MgCl_2_, 1 mM EGTA, 0.25-4.0 mM GTP, 22 °C). Km and Vmax values were determined by fitting the data points to a Hill-modified Michaelis-Menten equation (see Fig.4 for the data and curve fitting of each protein)

FtsZ species	K_m_ (mM)	V_max_ (GTP/FtsZ/min)	n
FtsZ (wild type)	1.3 ± 0.18	17 ± 1.9	2.2 ± 0.56
FtsZ84 (G105S)	3.6 ± 0.79	12 ± 3.6	3 ± 1.1
FtsZ84*F39L	0.5 ± 0.14	6 ± 1.1	1.2 ± 0.32
FtsZ84*M206I	0.5 ± 0.26	6 ± 1.9	1.5 ± 0.70
FtsZ84*V293I	0.57 ± 0.069	5.0 ± 0.83	4 ± 2.7

In contrast to K_m_, the maximum catalytic rates (V_max_) of FtsZ84 and the three intragenic suppressor mutants were significantly lower than wild-type FtsZ, suggesting the original G105S mutation interferes with catalysis independent of GTP binding. Wild-type FtsZ exhibited a V_max_ of 17 GTP/ FtsZ/min (Salvarelli et al. reported a V_max_ of 6.0 molecules GTP/ FtsZ/min and Sossong et al. reported 19.7 GTP/FtsZ/min [30, 31]). The V_max_ of FtsZ84 was reduced ~30 % to 12 GTP/ FtsZ/min (Table 3). The V_max_ values of all three FtsZ84* mutants were notably lower than FtsZ84, ranging from 5–6 molecules GTP/molecule FtsZ/min (Table 3). The lower V_max_ of the three intragenic suppressors may be a consequence of enhanced subunit-subunit interaction, which could interfere with nucleotide exchange and/or changes in the conformation of the active site that prevent optimal GTP hydrolysis.

Consistent with models for cooperative assembly in which GTP binding by one subunit leads to substrate binding effects on neighboring subunits, wild type FtsZ, as well as FtsZ84 and FtsZ84* V293I, exhibit positive cooperativity (*n >1*) (Table 4) [32]. FtsZ84*F39L and FtsZ84*M206I on the other hand do not exhibit the same degree of positive cooperativity (*n =* ~1).

**Table 4:** Strains and plasmids used in this study

Strain	Genotype	Reference/Source
MG1655 (PL2036)	*F- lambda-ilvG-rfb-50 rph-1*	
DRC14	MC4100 *ftsZ84*(ts) *leu*::Tn*10*	John Beckwith
*ftsZ84* (PL2452)	MG1655, *leu82*::Tn*10* *ftsZ84*(ts)	
ftsZ84*F39L (HA209)	MG1655, *leu82*::Tn*10* *ftsZ84* *F39L	This study
ftsZ84 *M206I (HA211)	MG1655, *leu82*::Tn*10* ftsZ84 *M206I	This study
ftsZ84 *V293I (HA213)	MG1655, *leu82*::Tn10 ftsZ84 *V293I	This study
HA223	MG1655, *leu82*::Tn*10* *ftsZ84*(ts), *pBAD-ftsW*	This study
*zapA* (HA235)	MG1655, *zapA*::*kan*	This study
*ftsZ84*, *zapA* (HA241)	*ftsZ84*, *zapA*::*kan*	This study
ftsZ84*F39L, *zapA* (HA267)	*ftsZ84* *F39L, *zapA*::*kan*	This study
ftsZ84*M206I, *zapA *(HA245)	*ftsZ84* *M206I,* zapA*::*kan*	This study
ftsZ84*V293I,* zapA* (HA251)	ftsZ84*V293I, *zapA*::*kan*	This study
HA255	MG1655, *pKG110-zapA*	This study
HA257	*ftsZ84*, *pKG110-zapA*	This study
CW206	MG1655, *pQE80-H6-zapA*	This study
HA364	*ftsZ84*, *pQE80-H6-zapA*	This study
Plasmid/Strain harboring plasmid	Genotype	Reference
pPJ2 (PL3142)	*pET21b(+)-ftsZ*	Buske and Levin, 2012
HA146	*pET21b(+)-ftsZ84*	This study
HA149	*pET21b(+) -ftsZ84* *F39L	This study
HA147	*pET21b(+)-ftsZ84* *M206I	This study
HA142	pET21b(+)-ftsZ84*V293I	This study
HA165	*pET21b(+)-ftsZ* *F39L	This study
HA157	*pET21b(+)*-*ftsZ* *M206I	This study
HA166	*pET21b(+)*-ftsZ*V293I	This study
PL3463	*pBAD24-ftsW*	This study
HA255	*pKG110-zapA*	Haeusser et al., 2014
HA364	*pQE80-H6-zapA*	Dajkovic et al., 2001

### FtsZ84*F39L acts synergistically with FtsZ84 (G105S) to restore GTPase activity

To gain an understanding of the impact of the three intragenic suppressor mutations on FtsZ assembly independent of FtsZ84 (G105S), we generated FtsZ constructs that are wild type at position 105, but harbor the individual suppressor mutations (FtsZ*) (Fig. 1b). Intriguingly, all three intragenic suppressor mutations reduced FtsZ mediated GTP hydrolysis independent of FtsZ84 (G105S) in our standard reaction conditions (5 μM FtsZ, 1 mM GTP, 30 °C)*.* FtsZ*F39L, in particular, reduced GTPase activity ~10-fold, indicating that the F39L mutation functions synergistically with the G015S mutation to restore GTPase activity. FtsZ*F39L hydrolyzed only 0.9 GTP/FtsZ/min, a rate comparable to FtsZ84 (0.47 GTP/FtsZ/min) and well below the FtsZ84*F39L hydrolysis rate of ~3 GTP/FtsZ/min (Table 2). FtsZ*M206I exhibited a modest ~20 % reduction in GTPase activity to 6 GTP/FtsZ/min and FtsZ*V293I had an ~65 % reduction to 3 GTP/FtsZ/min (Table 2).

### Intragenic suppressor mutations fail to restore FtsZ84 polymerization *in vitro*

A primary question was whether the enhanced subunit-subunit interaction, as evidenced by increased GTPase activity, was sufficient to support FtsZ84* polymer formation *in vitro*. To investigate this possibility, we examined the assembly dynamics of purified FtsZ84* proteins using two standard FtsZ assays: 90° angle light scattering and electron microscopy (EM). Based on the principle that polymers and polymer bundles will scatter more light than smaller structures such as FtsZ monomers, 90° angle light scattering is a standard assay for FtsZ assembly *in vitro* [10, 33–35]. A limitation of light scattering is its inability to distinguish between a large number of relatively small structures and a few very large structures. Electron microscopy (EM), in contrast, although not quantifiable, allows direct visualization of FtsZ ultrastructures and can distinguish even small polymers [10, 34, 35]. Together, these two approaches provide both a quantitative and a qualitative measure of FtsZ’s *in vitro* polymerization potential.

**Fig. 5:**
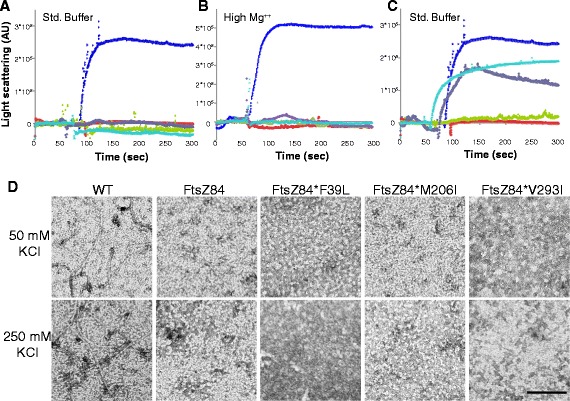
ftsZ84* suppressor mutations do not restore assembly *in vitro*. **a-b** 90° angle light scattering data from FtsZ (blue), FtsZ84 (red), FtsZ84*F39L (green), FtsZ84*M206I (purple), and FtsZ84*V293I (aqua) **a** 50 mM MES, pH 6.5, 50 mM KCl, *2.5 mM MgCl*_*2*_, 1 mM EGTA or **b** 50 mM MES, pH 6.5, 50 mM KCl, *10 mM MgCl*_*2*_, 1 mM EGTA (methods). After establishing baseline, assembly was initiated by the addition of 1 mM GTP. **c** 90° angle light scattering data from suppressor mutations alone (in the absence of *ftsZ84(G105S)*). FtsZ (blue), FtsZ84 (red), FtsZ*F39L (green), FtsZ*M206I (purple), and FtsZ*V293I (aqua) Reaction conditions same as in (**a**). **d** Electron micrographs of FtsZ assembled in 50 mM KCl or 250 mM KCl (50 mM MES, pH 6.5, 50 mM KCl, 2.5 mM MgCl_2_, 1 mM EGTA) Bar = 100 nm

Despite their ability to support cytokinetic ring formation *in vivo* under nonpermissive conditions, all three intragenic suppressor mutations failed to restore FtsZ84 polymerization *in vitro*. As previously reported, in standard buffer conditions (50 mM MES, pH 6.5, 50 mM KCl, 2.5 mM MgCl_2_, 1 mM EGTA) the addition of GTP stimulates assembly of wild-type *E. coli* FtsZ into single-stranded protofilaments readily visible by EM (Fig. 5d) [4, 36]. In contrast, FtsZ84, FtsZ84*F39L, FtsZ84*M206I, and FtsZ84*V293I all failed to assemble *in vitro* following the addition of GTP (Fig. 5). The light scattering signal remained near baseline and even decreased slightly for all 3 FtsZ84* mutant proteins and no FtsZ polymers were evident in EM images (Fig. 5a,d). Notably, the appearance of FtsZ84 and FtsZ84* assembly reactions by EM is strikingly similar, suggesting that any differences in assembly that might be present are too subtle to visualize, even using this high-resolution technique. We should note that a more sensitive technique such as dynamic light scattering might reveal subtle differences in *in vitro* assembly that more standard approaches are unable to detect.

To determine if the defect in assembly of FtsZ84 and FtsZ84* was due, at least in part, to loss of lateral interaction potential or if it was primary due a deficiency in longitudinal subunit interactions, we increased the concentration of magnesium from 2.5 mM to 10 mM to stimulate lateral interactions between FtsZ protofilaments [33]. While the mechanism is likely different – charge shielding versus cross-linking—*in vitro* the addition of divalent cations largely mimics the impact of FtsZ-modulatory proteins that enhance lateral interactions to bundle protofilaments [33, 37]. Consistent with a defect in longitudinal interactions, the addition of 10 mM [Mg^++^] had no impact on assembly of FtsZ84 or the three FtsZ84* mutant proteins in light scattering assays (Fig. 5). Increasing the potassium ion levels from 50 mM to 250 mM, which has been reported to stimulate assembly of wild - type FtsZ proposedly through increasing stability of the GTP-bound FtsZ dimer [33, 38], also had no detectable impact on assembly of mutant FtsZ protein (Fig. 5d). Although pH has been reported to impact FtsZ, assembly, we elected not to vary pH, as the conditions we employed (pH 6.5) are the most permissive of the buffer conditions standardly employed for FtsZ analysis [2].

Light scattering indicates that the impact of the F39L, M206I and V293I mutations on assembly of otherwise wild-type FtsZ roughly parallels their impact on GTP hydrolysis. Under the standard reaction conditions described above, FtsZ*F39L protein assembled to levels only slightly above baseline, consistent with its ~10-fold reduction in GTP hydrolysis, while FtsZ*M206I and FtsZ*V293I assembled to levels ~65 % of wild type, consistent with their more robust GTPase activity (Fig. 5c).

### Intragenic suppressors of *ftsZ84* rely on other components of the cell division machinery for growth and division under nonpermissive conditions

Based on their inability to form stable protofilaments *in vitro*, we speculated that the all three FtsZ84*s as well as their FtsZ84 parent protein were dependent on modulatory proteins. One candidate protein ZapA has previously been shown to stabilize FtsZ protofilaments *in vitro* and *in vivo* [17, 39]. To test if ZapA is helping stabilize FtsZ84* under nonpermissive conditions, we examined the impact of deleting *zapA* on the viability of *ftsZ84* and ftsZ84* mutants under both permissive and nonpermissive conditions.

**Fig. 6:**
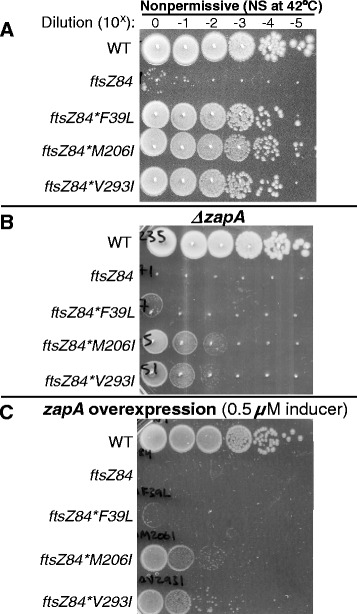
*zapA* deletions reduce ftsZ84* viability under nonpermissive conditions. **a** Plating efficiency of WT, *ftsZ84,* and ftsZ84* strains when grown in permissive conditions and plated onto nonpermissive conditions. **b** Plating efficiency assays of *zapA* deletions in wild-type, *ftsZ84*, and ftsZ84* suppressor strains. Tenfold dilutions of cells cultured in permissive conditions were plated under nonpermissive conditions. **c** *zapA* overexpression from a sodium salicylate inducible *zapA* plasmid (pKG110-*zapA*) does not restore viability to *ftsZ84* and makes ftsZ84* suppressor strains more sensitive when plated under nonpermissive conditions with 0.5 μM of sodium salicylate. Identical results were obtained when using the IPTG inducible *zapA* vector (pQE80-H6-*zapA*) in *ftsZ84* (see Additional file 1). **b** and **c** These experiments were performed at least 3 times with equivalent results

Although loss of *zapA* had no impact on colony forming ability under permissive conditions, *zapA* was required for full ftsZ84* mutant viability under nonpermissive conditions (LB no salt at 42 °C) (Fig. 6). Under nonpermissive conditions, the *zapA* deletion reduced the colony forming ability of ftsZ84*F39L cells ~1,000-10,000-fold and the colony forming ability of ftsZ84*M206I and the ftsZ84*V293I ~100-fold (Fig. 6b).

Since ZapA has been previously been reported to stabilize FtsZ protofilaments *in vitro* [17], we next tested whether overexpressing *zapA* would restore colony-forming ability to *ftsZ84* cells under semi-permissive and nonpermissive conditions. Surprisingly, overexpression of *zapA* from two different inducible/repressible promoters (a sodium salicylate inducible construct, the gift of Bill Margolin, and an IPTG-inducible construct, the gift of Alex Dajkovic) failed to restore growth to *ftsZ84* strains under nonpermissive conditions, suggesting that ZapA is not the sole determinant of FtsZ84* assembly potential *in vivo* (Fig. 6c, Additional file 1: Figure S1). In fact, *zapA* expression from a strong plasmid based promoter significantly reduced the ability of all three ftsZ84* mutations to suppress *ftsZ84* heat sensitivity (Fig. 6c). In particular, *zapA* overexpression reduced colony formation in ftsZ84*F39L by 10,000-fold and ftsZ84*M206I and ftsZ84*V293I by ~100-fold under non-permissive conditions (Fig. 6c).

## Discussion

**Fig. 7:**
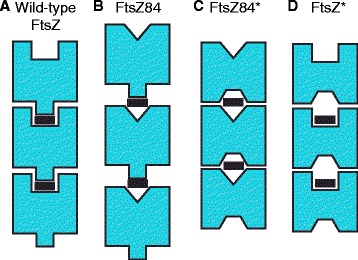
FtsZ84*M206I and FtsZ84*V293I mutations compensate FtsZ84 to increase GTPase activity and longitudinal subunit interactions. **a** Wild-type FtsZ efficiently binds to and hydrolyzes GTP (black rectangle). This promotes subunit interactions and polymerization. **b** FtsZ84 contains a mutation in the GTP binding site that reduces GTP binding and hydrolysis, thereby weakening FtsZ dimerization and subunit-subunit assembly. **c** FtsZ84*M206I and FtsZ84*V293I mutations compensate for FtsZ84’s GTP binding defect and help to restore subunit-subunit interactions. **d** Introducing FtsZ84*M206I and FtsZ84*V293I into wild-type FtsZ reduces GTPase activity and assembly

Our data support a model in which disruption of interactions between FtsZ monomers at the longitudinal subunit interface is the underlying cause of *ftsZ84* conditional lethality (Fig. 7). Wild-type FtsZ is optimized for GTP binding and hydrolysis (Fig. 7a). Like tubulin, GTP binding leads to a conformational change that increases subunit-subunit affinity and promotes dimerization and protofilament formation [2]. As indicated by the increased K_m_ of FtsZ84 as compared to wild-type FtsZ, the (G105S) mutation in the GTP binding site interferes with GTP binding (Fig. 7b). Based on our finding that the FtsZ84* mutants restore GTP binding as estimated by K_m_, we propose the intragenic suppressor mutations increase subunit-subunit affinity and promote nucleotide binding by trapping GTP between in the active site between FtsZ subunits (Fig. 7c).

FtsZ84’s V_max_ is approximately 70 % that of wild type FtsZ (Table 3) suggesting that FtsZ84 not only has a reduced affinity for GTP, but is also defective for catalysis. The intragenic suppressor mutations further reduce the FtsZ’s catalytic rate from to ~ half the rate of FtsZ84. This reduction in V_max_ may be due to (1) a conformational change in the protein that alters GTP binding such that it is no longer in the optimal orientation for efficient hydrolysis and/or (2) an increased subunit-subunit interaction that promotes GTP retention at the subunit interface, limiting nucleotide exchange.

Enzyme kinetics suggest positive cooperativity with regard to GTP binding for wild-type FtsZ, FtsZ84 and FtsZ84*V293I (*n >*1) (Table 4) consistent with the model for cooperative assembly suggested by Miraldi and Romberg [32]. However, the significance of this observation in unclear due to the challenge of assessing the value of *n* for a polymerizing enzyme such as FtsZ that is reported to exchange nucleotide in the polymer itself [40, 41]. Moreover, neither FtsZ84*F39L nor FtsZ84*M206I exhibit positive cooperativity (*n = ~*1 for both). Further complicating interpretation is data from Salvarelli et al. suggesting that GTPase active sites are independent in FtsZ filaments [31]*.* Regardless, further work is needed to resolve the impact of nucleotide binding on FtsZ conformation and investigate the potential cooperative nature of FtsZ filaments.

The location of M206I in the T7 catalytic loop and V293I along the subunit-subunit interface suggests that both mutations directly enhance interactions between subunits and thus promote GTP retention at the active site (Fig. 7c). Consistent with this model, the M206I and V293I single mutants exhibited only moderate reductions in GTPase activity (Table 2, Fig. 7d). While exhibiting lower GTPase activity, FtsZ*M206I was not statistically different from wild type. FtsZ*V293I exhibited ~2-fold less GTPase activity relative to its wild-type parent. On the other hand, based on its location in the interior of the FtsZ core domain (Fig. 1), we favor the idea that the F39L defect suppresses *ftsZ84* heat sensitivity by counteracting the impact of the G105S substitution through changes in the conformation of the GTP binding site. This model is consistent with FtsZ*F39L’s significantly impaired GTPase activity in the absence of G105S.

We speculate that modulatory proteins that enhance FtsZ assembly are the underlying cause of the dichotomy in assembly potential exhibited by FtsZ84 and FtsZ84* *in vitro* and *in vivo.* FtsZ ring formation is generally thought to be a two-step process involving first the formation of transient, single stranded FtsZ polymers that are subsequently stabilized through lateral interactions mediated by a host of modulatory proteins [42]. The dynamic nature of the FtsZ ring means that maintenance of the ring similarly requires the both strong subunit-subunit interactions and subsequent stabilization by modulatory proteins [43]. Supporting the idea that defects in FtsZ assembly can be compensated for by intragenic mutations, the Margolin laboratory recently identified a mutation in *E. coli* FtsZ (L169W) that bypasses the requirement for the normally essential bundling protein ZipA and confers resistance to defects in other components of the division machinery [44].

Applying this model to *ftsZ84* cells under nonpermissive conditions, reduced subunit affinity would disrupt the early steps in FtsZ84 assembly limiting the formation of single stranded polymers and impacting downstream interactions with stabilizing modulatory proteins. Consistent with this model, shifting *ftsZ84* mutants to nonpermissive conditions prevents the formation of new FtsZ rings and leads to the rapid disassembly of all extant FtsZ rings [45].

Based on our analysis of the FtsZ84* intragenic suppressor mutations, we propose the restoration of subunit-subunit affinity is sufficient to support the transient assembly of FtsZ84* polymers and subsequent interaction with modulatory proteins, ultimately permitting formation of a functional cytokinetic ring under nonpermissive conditions. *In vitro*, however, the absence of modulatory proteins precludes both FtsZ84 and FtsZ84* from forming polymers stable enough to detect by either 90° angle light scattering or EM. Consistent with this model, the loss of ZapA, which stabilizes FtsZ protofilaments, severely reduces the viability of FtsZ84 and FtsZ84* mutants under nonpermissive conditions (Fig. 6b). Elucidation of the molecular mechanisms by which modulatory proteins promote FtsZ assembly and enhance the integrity of the FtsZ ring should clarify the apparent discrepancy between the assembly dynamics FtsZ84 and FtsZ84* *in vitro* and *in vivo*.

## Conclusions

This study suggests a mechanism by which FtsZ84, a polymerizing enzyme with defective catalysis, gains function through increased substrate binding and enhanced subunit-subunit interactions. Although kinetic analysis clearly indicates a significant enhancement in substrate interaction (Fig. 5), this increase in Km is insufficient to restore assembly to FtsZ84* *in vitro*, although all three suppressor mutants supported assembly *in vivo* under both permissive and nonpermissive conditions. In theory, defects in assembly of other polymerizing enzymes might also be compensated for by increases in subunit affinity. While this increased binding is evident through enzyme kinetics, it does not restore assembly to FtsZ84* in common *in vitro* assembly reactions, although these proteins can assemble *in vivo* at both permissive and nonpermissive conditions.

By illuminating the disconnect between *in vitro* and *in vivo* assembly, this study highlights the value of pairing biochemistry with genetics and cytology in order to draw biologically meaningful conclusions. While light scattering and EM are invaluable for evaluating gross changes in FtsZ assembly, understanding FtsZ assembly dynamics *in vivo* will ultimately require the development of super resolution approaches for imaging of cytokinetic ring formation in real time as well as approaches to reconstitute FtsZ assembly *in vitro* in the context of its full complement of modulatory proteins.

## Methods

### General methods and strain construction

All *E. coli* strains are derivatives of MG1655 and are listed in Table 4.

The *ftsZ84* allele from DRC14 (the gift of Debabrata RayChaudhuri) was moved into MG1655 via P1 phage transduction to create PL2452 (*ftsZ84*) [18, 46]. Cells were grown in permissive conditions in lysogeny-broth (LB) Miller (10 g/L tryptone, 5 g/L yeast extract, 10 g/L NaCl) at 30 °C or nonpermissive conditions of 42 °C in LB-no salt media (10 g/L tryptone, 5 g/L yeast extract) where indicated.

Alleles were moved between strains by P1 phage transduction [18]. Cloning, transformations and phage transduction were carried out under standard conditions [18, 47]. Ampicillin (100 μg/ml), tetracycline (12.5 μg/ml) and arabinose (0.5 %) were used as needed for maintenance of episomal DNA, selection of linked alleles following genetic exchange, and induction of gene expression, respectively.

### Whole genome sequencing

The genomes of our *ftsZ84* (PL2452) strain and the parental MG1655 strain (PL2036) were subjected to both Illumina and 454 sequencing courtesy of The Genome Institute at Washington University in St. Louis. Allelic differences between the wild-type MG1655 parental strain (PL2036) and *ftsZ84* (PL2452) were confirmed by both Illumina and 454 sequencing.

### Identification of intragenic suppressors of ftsZ84

*ftsZ84* cells were grown to mid-exponential phase (OD_600_ 0.2-0.5) in permissive conditions (30 °C, LB 1 % NaCl). 1 ml of cell culture was removed, washed once in LB - no salt media and plated onto LB-no salt plates at 42 °C. Colonies were verified for temperature resistance and struck onto LB-no salt plates at 42 °C. Suppressors were analyzed for linkage to *ftsZ84* using P1 phage transduction [18] onto LB plates containing tetracycline and then transductants were patched onto nonpermissive conditions to determine whether temperature resistance was linked to *ftsZ84*. The *ftsZ84* allele of linked mutations was amplified by polymerase chain reaction to determine if the suppressor mutation was intragenic. The *ftsZ84* alleles containing intragenic suppressor mutations were moved by P1 transduction to our wild-type MG1655 background.

### EMS mutagenesis

For ethyl methanesulfonate (EMS) mutagenesis, cells were grown to mid-exponential phase (OD_600_ 0.2-0.5) in permissive conditions. Cells were then treated with 25 μg/ml EMS for 45 minutes at 30 degrees (in LB-Miller 1 % salt media) prior to 3 washes in LB-no salt media and plating on LB-no salt plates. Mutant verification was carried out as above.

### Growth rate determination

Strains were grown under permissive conditions to mid-exponential phase (OD_600_ 0.2-0.5). Cells were pelleted and washed once with LB-no salt media and back-diluted to a calculated OD_600_ of 0.025 in prewarmed (42 °C) LB-no salt (nonpermissive conditions) or LB-1 % NaCl at 30 °C (permissive conditions). Absorbance (OD_600_) was measured every 30 minutes post back-dilution for 6 hours. The doubling time during exponential phase was calculated using www.doubling-time.com.

### Plating efficiency assay

Cells were grown to mid-exponential phase (OD_600_ 0.2-0.5) under permissive conditions. Cells were pelleted and washed once in LB-no salt media. Cells were resuspended in LB-no salt medium to a calculated OD_600_ of 0.2. 10 μl of tenfold serial dilutions were spotted on LB agar and LB-no salt agar at 30 °C and 45 °C.

### Fluorescence microscopy

For imaging of cells grown at 42 °C in LB-no salt media, cells were first cultured in LB 1 % NaCl at 30 °C to an OD_600_ of 0.2-0.5, washed once in LB-no salt media, and back-diluted to a calculated OD_600_ of 0.05 in prewarmed LB-no salt media. Cells were then cultured under nonpermissive conditions for 75 minutes (~3 wild-type mass doublings).

### Live cell imaging

For live cell imaging, the membrane stain FM4-64 was added to the cell sample at a final concentration of 1 μg/ml and incubated for 5 minutes. The cells were spotted on agarose pads containing 1 % agarose in 1X phosphate buffered saline (PBS). Images were captured within 10 minutes of placing the cells on the slides. Cell length was calculated by measuring the distance of the cells from pole to pole. Only cells that were completely visible in the field were measured.

### Immunofluorescence microscopy

Immunofluorescence microscopy was performed as previously described [26, 48]. Briefly, cells were fixed through a glutaraldehyde-paraformaldehyde fixation. To prepare the fix, 6.25 μl of 8 % glutaraldehyde was added to 1 ml of 16 % paraformaldehyde on ice. Immediately before fixation, 100 μl of the fix was added to 20 μl of 1 M NaPO_4_ pH 7.4. 500 μl of the cell culture was then added and the tubes were inverted. The samples were then incubated for 15 minutes at room temperature followed by 30 minutes on ice. Fixed cells were pelleted at 14,000 x g in a microcentrifuge, washed 3 times with 1 mL of 1X PBS, resuspended in GTE (50 mM glucose, 25 mM Tris, 10 mM EDTA, pH 8.0) to a final OD_600_ of ~0.2 and stored at 4 °C. To stain cells for immunofluorescence, samples were affixed to poly-l-lysine coated slides and then treated with 2 μg/ml lysozyme for 2 minutes to weaken the cell wall. Staining was as follows: cells were blocked with 2 % bovine serum albumin (BSA) for 15 minutes, treated with 1:500 rabbit anti FtsZ antibody in 2 % BSA at 37 °C for 30 minutes, washed 8+ times with 1X PBS, and finally treated with 1:500 alexafluor 488-conjugated goat anti rabbit secondary antibody and tetrarhodamine isothiocyanate-conjugated wheat germ agglutinin (to stain cell walls) for 30 minutes at room temperature in the dark. Cells were visualized using an Olympus BX51 microscope equipped with a 100X brightfield objective and an Orca ER camera (Hammatsu). Length-per-ring ratios were calculated by measuring the total length of cells and counting FtsZ rings using Openlabs image analysis software.

### Quantitative immunoblotting

Quantitative immunoblotting was performed essentially as described in Weart and Levin, 2003 [4], with the following modifications. To concentrate cells, 20 ml of culture at an OD_600_ of 0.2 was pelleted and resuspended in 500 μl PBS in tubes containing ~70 mg silica beads. Cell lysates were prepared by bead beating in a FastPrep (MD Biomedical) machine for 2 pulses of 20 seconds each at 6.0 m/s. Gel loading was normalized to the sampling OD_600_. Ponceau S staining after the transfer was used as a loading control to normalize FtsZ staining to total protein. FtsZ was visualized using a 1:5,000 dilution of affinity purified rabbit antisera raised against *E. coli* FtsZ, the kind gift of David Weiss, and a secondary antibody conjugated to Horse Radish Peroxidase (Jackson ImmunoResearch Laboratories, West Grove, Pa). Quantification of FtsZ-bands and Ponceau staining was performed with ImageJ software. All quantifications were performed in the linear range of detection for the samples and antibodies. Note that using this approach we are able to reliably detect differences in intracellular FtsZ concentration between 10 % and 15 % [26].

### Protein purification

FtsZ variants were cloned in the pET-21b(+) expression vector using the *E. coli* strain AG1111. The resulting purified plasmids were freshly transformed into C41(DE3) cells [10] and consequently used for the expression of proteins. Briefly, 1 liter of LB medium was inoculated 1:100 with an overnight culture started from a single colony. Cells were grown at 37 °C until OD_600_ was ~0.6 after which *ftsZ* expression was induced with 1 mM IPTG (isopropyl 1-thio-β-D-galactopyranoside). Cells were grown for an additional 4 hours at 37 °C and then cells were harvested by centrifugation and pellets were stored at −80 °C. To purify FtsZ, frozen cell pellets were thawed on ice and resuspended in 30 mL induction buffer (50 mM Tris, pH 8.8, 100 mM NaCl, 1 mM EDTA) with 1 mM AEBSF (4-(2-Aminoethyl) benzenesulfonyl fluoride hydrochloride). Proteins were subjected to two rounds of lysis on a French press at 10,000 psi and the lysate clarified by spinning at 160,000xg for 45 minutes at 4 °C.

FtsZ was precipitated from the supernatant with ammonium sulfate. In all cases, 0.25 of the volume of saturated ammonium sulfate was added to bring solution to a final concentration 20 %. Samples were incubated on ice for 20 minutes and spun at 10,000xg for 10 minutes at 4 °C. FtsZ84, FtsZ84*F39L, FtsZ84*M206I, and FtsZ84*V293I all precipitated in the first ammonium sulfate cut (20 % final concentration ammonium sulfate). For wild-type FtsZ, the supernatant was transferred to a new tube and 0.14 times the volume of saturated ammonium sulfate was added to bring the final concentration of ammonium sulfate to 30 %. The sample was incubated on ice for 20 minutes and spun at 10,000xg for 10 minutes at 4 °C.

In all cases, pellets containing the bulk of the FtsZ protein were resuspended in low salt anion exchange buffer (50 mM Tris, pH 8.5, 50 mM KCl, 1 mM EGTA, 10 % sucrose) and further purified on a MT-20 column manually packed with UNOsphere™Q media with a linear gradient of 50–500 mM KCl in 50 mM Tris, pH 8.5, 1 mM EGTA, 10 % sucrose. Peak fractions were analyzed by SDS-page, pooled, and dialyzed overnight in 1 liter of FtsZ dialysis buffer, pH 7.5 (50 mM HEPES, pH 7.5, 50 mM KCl, 2.5 mM MgCl_2_, 1 mM EGTA, 10 % sucrose). FtsZ84 and FtsZ84* proteins were released from the column in a wider concentration of KCl than FtsZ and the FtsZ* proteins. Protein preparations were concentrated using PEG, aliquoted, flash frozen with liquid nitrogen, and stored at −80 °C.

### 90° angle light scattering assay

Light scattering assays were performed as previously described [10, 29] using a DM-45 spectrofluorimeter (Olis). Readings were taken every 0.25 seconds and a baseline reading was established for 60–100 seconds before addition of 1 mM GTP. Assembly reactions contained 5 μM FtsZ in standard assembly buffer (50 mM MES, pH 6.5, 50 mM KCl, 2.5 mM MgCl_2_, 1 mM EGTA) or in a high Mg^++^ buffer that instead contained 10 mM MgCl_2_. Data were collected by SpectralWorks (Olis) and exported into Microsoft Excel for processing.

### Electron microscopy

Electron microscopy was performed as described [1, 2, 49]. FtsZ was assembled with GTP as for light scattering in the standard buffer (50 mM MES, pH 6.5, 50 mM KCl, 2.5 mM MgCl_2_, 1 mM EGTA) or a buffer that instead had 250 mM KCl. 5 μM FtsZ was used for all in vitro assembly experiments. Samples were visualized using a JEOL 1200EX transmission electron microscope.

### GTPase assay

GTPase activity was measured as previously described [2, 10], using the continuous, regenerative coupled GTPase assay of Ingerman and Nunnari [2, 8, 50]. Briefly, assays were conducted in buffer conditions identical to the standard buffer used for light scattering with 5 μM FtsZ, 1 mM GTP, 1 mM phosphoenolpyruvate, 250 μM NADH, 80 units/ml lactose dehydrogenase and 80 units/ml pyruvate kinase. GTPase assays were performed at 30 °C. We used the linear decline of absorbance for NADH at 340 nm for 3 min in a quartz cuvette (1-cm path length) using a SPECTRAmax Plus spectrophotometer (Molecular Devices). The raw data of absorbance per minute was converted to activity using the extinction coefficient for NADH of 6220 M^−1^ cm^-1^. GTPase data are the average of 3–5 independent experiments.

### Kinetics curves

Kinetics curves were generated by performing GTPase assays as in the previous paragraph while varying the GTP concentrations from 0.1 to 6 mM (all kinetics data obtained at 22 °C using an Eon spectrophotometer (BioTek). At least 5 GTP concentrations were used per FtsZ mutant and values used to calculate the K_m_ and V_max_ were the average of at least 3 replicates per GTP concentration. The K_m_ and V_max_ were calculated using Sigma Plot software. The best fit for the data was obtained using the Hill-modified Michaelis-Menten equation: V_0_ = V_max_[S]^n^/(K_m_^n^ + [S]^n^).

### *zapA* deletion and overexpression experiments

*zapA* deletions and overexpression strains were generated utilizing standard genetic procedures (transduction and transformation) discussed above [18]. The *zapA* deletion strain from the Keio collection was acquired through the *E. coli* stock center at Yale and was used as a donor for phage transduction into our recipient wild-type, *ftsZ84,* and ftsZ84* strains. The sodium salicylate inducible *zapA* plasmid (pKG110-*zapA*) (gift of Bill Margolin) and the IPTG inducible *zapA* plasmid (pQE80-H6-*zapA*) (gift of Alex Dajkovic) were transformed into the wild-type and *ftsZ84* background strains [17, 51]. For the plating efficiency assays (see above), all strains were grown in LB-1 % NaCl to an OD_600_ of 0.2 and plated onto LB-no salt plates (*zapA* deletion strains) or LB-no salt ampicillin plates with 0, 0.1 μM, or 1.0 μM sodium salicylate.

## Availability of supporting data

All supporting data are included in the following additional file to the manuscript:

Additional file 1, Adobe Acrobat .pdf format, *zapA* overexpression does not restore colony formation to *ftsZ84* cells under nonpermissive conditions, this file shows colony forming data of *ftsZ84* cells when the *zapA* gene is induced.

## Ethics statement

Ethics approval was not required for this study, which utilized only bacteria and did not involve humans, human data or animals.

## Additional file

Additional file 1:
***zapA*** **overexpression does not restore colony formation to** ***ftsZ84*** **cells under nonpermissive conditions.** (PDF 807 kb)

## Abbreviations

LB: Lysogeny broth
OD: Optical density
L/R: Length per ring
EMS: Ethylmethanesulfonate
EM: Electron microscopy
