# Supplementary material for: Mutations in the bacterial cell division protein FtsZ highlight the role of GTP binding and longitudinal subunit interactions in assembly and function
Source: BMC Microbiol. 2015 Oct 13;15:209. doi: 10.1186/s12866-015-0544-z (PMC4603965; doi:10.1186/s12866-015-0544-z)
Supplement: Additional file 1: — zapA overexpression does not restore colony formation to ftsZ84 cells under nonpermissive conditions. (PDF 807 kb) [file 12866_2015_544_MOESM1_ESM.pdf]

Additional File 2: *zapA* overexpression does not restore colony formation to *ftsZ84* cells under nonpermissive conditions

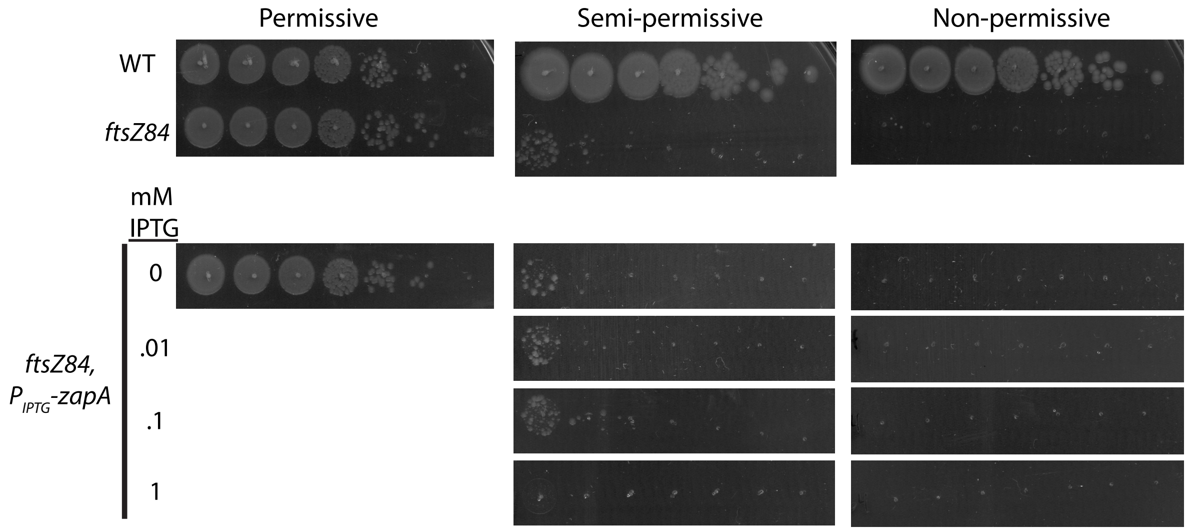

Cells were grown to an OD_{600} of 0.2 in permissive conditions without inducer (LB-1%NaCl, 30°C). Cells were serially diluted 10-fold and 10 μl of each dilution were plated onto permissive (LB-1% NaCl, 30°C), semi-permissive (LB-no salt, 37°C), or nonpermissive (LB-no salt, 42°C) conditions with varying concentrations of IPTG
